# The Treatment of 102 Carriers of Entamoeba Histolytica with Emetine Bismuth Iodide

**Published:** 1918-05

**Authors:** 


***The Treatment of 102 Carriers of Entamoeba Histolytica with Emetine Bismuth Iodide.*** By Capt. W. Waddell, R. A. M. C., Capt. C. Banks, R.A.M.C., H. Watson, and W. O. Redman King. *Journal R. A. M. C.*, March, 1918.

This report covers 102 men who were discovered to be carriers of Entamoeba histolytica in the course of ordinary routine examinations of faeces at a dysentery convalescent hospital. The treatment was carried out under the supervision of one medical officer and the specimens examined on the spot. None of the cases were found to be carriers of dysentery bacilli.

The form of the drug used was a keratin-coated tabloid containing one grain of emetine bismuthous iodide and as a rule the daily dose, consisting of three grains, was given to each man after the mid-day meal.

It was found that emetine bismuthous iodide was much more effective than emetine hydrochloride but that 20 per cent. of failures occurred. The drug had extremely irritating effects on, the men and there was no real tolerance, though, as time went on, some of them were not so often sick. There was definite failure to cure in nineteen cases, in all of which undoubted examples of E. histolyticus were found, often in large numbers, on at least two occasions after treatment; the authors consider that the failures may have been due in part to the form of drug used. They do not believe there were many relapses later than those discovered and that, as several of the cases had only 8 or 9 doses instead of the 12 usually given, less than 36 grains may be effective. The drug is without appreciable effect upon the intestinal flagellates, but has an effect, usually temporary, on E. coli.

